# CD154: An Immunoinflammatory Mediator in Systemic Lupus Erythematosus and Rheumatoid Arthritis

**DOI:** 10.1155/2012/490148

**Published:** 2011-10-24

**Authors:** Nada Alaaeddine, Ghada S. Hassan, Daniel Yacoub, Walid Mourad

**Affiliations:** ^1^Department of Pathology, Faculty of Medicine, P.O. Box 11-5076, St Joseph University, Beirut, Lebanon; ^2^Laboratoire d'Immunologie Cellulaire et Moléculaire, Centre Hospitalier de l'Université de Montréal, Hôpital St-Luc, Montréal, QC, Canada H2X 1P1

## Abstract

Systemic lupus erythematosus and rheumatoid arthritis are two major chronic inflammatory autoimmune diseases with significant prevalence rates among the population. Although the etiology of these diseases remains unresolved, several evidences support the key role of CD154/CD40 interactions in initiating and/or propagating these diseases. The discovery of new receptors (**α**IIb**β**3, **α**5**β**1, and **α**M**β**2) for CD154 has expanded our understanding about the precise role of this critical immune mediator in the physiopathology of chronic inflammatory autoimmune diseases in general, and in systemic lupus erythematosus and rheumatoid arthritis in particular. This paper presents an overview of the interaction of CD154 with its various receptors and outlines its role in the pathogenesis of systemic lupus erythematosus and rheumatoid arthritis. Moreover, the potential usefulness of various CD154-interfering agents in the treatment and prevention of these diseases is also discussed.

## 1. Introduction

Over the last decade, considerable new insights into the pathogenesis of systemic lupus erythematosus (SLE) and rheumatoid arthritis (RA) have been provided. SLE involves the production of autoantibodies against many self antigens, and the formation of immune complexes affecting many tissues and organs. RA, on the other hand, mainly implicates joints and articulations, where chronic inflammation of the synovial tissues leads to destructive tissue damage. Mechanistically, interactions between the extracellular matrix, cell surface receptors, and soluble mediators are at the center of the inflammatory response that takes place in these autoimmune diseases. Indeed, most of the new biological therapies currently in use or in clinical development are directed at membrane associated targets or their ligands. In this matter, CD154, in its membrane-bound or soluble form, was shown to be an important modulator of immunoinflammatory events in autoimmune diseases [1]. Here we review the major and most recent findings on the role of CD154 in various inflammatory autoimmune diseases, while focusing in particular on SLE and RA. Accumulating knowledge will allow us to better understand the CD154 axis in disease states, thereby, facilitating the design of new preclinical approaches and more efficient therapies for the treatment of CD154-associated pathologies.

## 2. CD154

CD154, also known as CD40 ligand (CD40L), previously referred to as gp39, TRAP, or TBAM [2–4], is a 39 kDa type II membrane glycoprotein of the TNF family [5]. Located on the long arm of the X chromosome, region q26.3–q27.1, the human CD154 gene is composed of five exons and four introns. It encodes a polypeptide of 261 amino acids (aa), consisting of a 215 aa extracellular domain, a 24 aa transmembrane region, and a 22 aa cytoplasmic tail [6–9]. Like other members of the TNF-family, CD154 forms a trimeric structure and promotes as such trimerization of the receptor, namely, CD40 [10]. The CD154/CD40 interaction is stabilized by charged residues, namely, the basic chains on CD154 and the acidic ones on CD40 [11, 12]. It has been reported that the CD154/CD40 interaction is required for the proteolysis of membrane-bound CD154 and the subsequent release of soluble CD154 (sCD154) by activated platelets [13]. Soluble CD154 is an 18 kDa fragment comprised of residues 113–261 of the membrane-bound CD154 molecule and remains a functional trimer retaining its ability to bind receptors. Indeed, sCD154 shares similar activities with the membrane bound form and has been shown to be associated with many autoimmune diseases [14]. The CD154 homotrimer is nonconstitutively expressed on different cell types, including activated T lymphocytes, basophils, eosinophils, monocytes, macrophages, natural killer cells, B lymphocytes, platelets, dendritic cells, as well as endothelial, smooth muscle, and epithelial cells [14]. 

Accumulating evidence now indicates that CD154 can bind to receptors other than CD40, namely, the integrins *α*IIb*β*3, *α*5*β*1, and *α*M*β*2 [15–19]. 

## 3. CD154 Receptors

### 3.1. CD40

CD40 is a 48 kDa type I membrane glycoprotein and member of the tumor necrosis factor receptor (TNFR) super family [5]. The human CD40 gene was localized to the long arm of chromosome 20 along 2q12–q13.2. [20], and Tone et al. have showed that it contains 9 exons [21]. Transcription of the gene yields a phosphoprotein of 277 amino acids composed of a signal peptide, an extracellular domain including 22 cysteine residues implicated in its ligand binding, two potential N-linked glycosylation sites, a transmembrane region central for its translocation and clustering into lipid rafts microdomains, and a cytoplasmic region [11, 22–25].

The precise structure by which CD40 is found on the cell surface is still a matter of controversy. Despite the initial suggestion that CD40 exists as a dimer that is being trimerized upon CD154 ligation, more recent studies describe CD40 as a constitutively preassembled trimer [26]. Moreover, we have shown that upon CD154 ligation, CD40 can oligomerize into dimers between cysteine residues 238 located in the intracellular region of the molecule. Interestingly, this dimerization appears essential for phosphoinositide-3 kinase (PI-3K) activation and the subsequent activation of B7.2, as well as the production of IL-8 in B cells. Recently, the CD154/CD40 complex has been described as a trimeric CD154 molecule interacting with a CD40 dimer, as the third CD40 molecule is pushed out of the complex by charged residues [12]. Hence, additional studies are required for further clarification of the exact structure of CD40 on the cell surface. 

**Figure 1:**
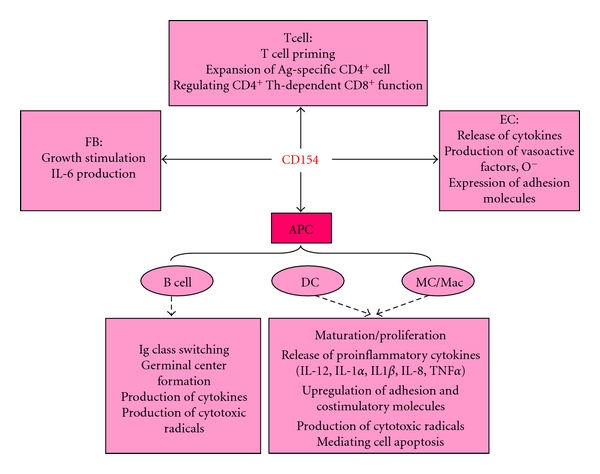
Biological function of the CD154/CD40 interaction. CD154 mediates numerous inflammatory functions on a wide variety of cell types by interacting with its classical CD40 receptor. These include T cell priming, B cell-dependent Ig class switching and germinal center formation, cell proliferation, regulation of apoptosis, release of proinflammatory cytokines, upregulation of adhesion molecules and costimulatory molecules, and production of cytotoxic radicals. FB: fibroblast, EC: endothelial cell, APC: antigen presenting cells, DC: dendritic cell, MC/Mac: monocyte/macrophage.

CD40 is constitutively expressed on a wide variety of cells including B cells, dendritic cells, macrophages, endothelial cells, fibroblasts, platelets, osteoblasts, smooth muscle cells, neurons, pancreatic beta cells, and ductal cells. CD40 was also shown to be induced by various cytokines such as TNF-*α* and interleukin-1*β* (IL-1*β*) in a variety of cell types [9, 27]. Upon its engagement, CD40 induces a pattern of gene expression depending on the particular cell type involved. On B cells, it is critical for survival and proliferation, isotype switching, germinal center formation, memory generation, and production of numerous cytokines and chemokines such as IL-1, IL-6, IL-8, IL-10, IL-12, TNF-*α*, and cytotoxic radicals. On T cells, CD40 can influence cell priming and cell-mediated effector functions including macrophage and natural killer cell activation [14]. On vascular endothelial cells, CD40 stimulates cytokine production, upregulation of adhesion molecules, release of superoxide anions, and expression of cyclooxygenase-2 [1, 28, 29]. On dendritic cells, monocytes and macrophages, CD40 stimulates the production of cytokines such as TNF and contributes to the rescue of circulating monocytes from apoptosis [14] (Figure 1). The extensive distribution and various inflammatory functions of the CD154/CD40 axis explain its involvement in the pathogenesis of many autoimmune diseases.

### 3.2. *α*IIb*β*3

**Figure 2:**
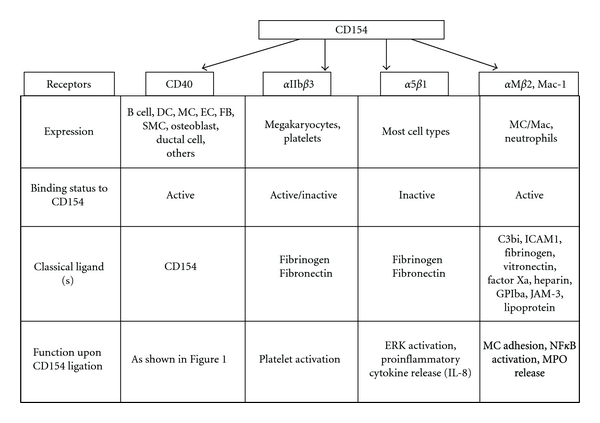
Structural and functional characteristics of CD154 receptors. Four receptors for CD154 have been identified, namely, CD40, *α*IIb*β*3, *α*5*β*1, and *α*M*β*2. These receptors are found on various cell types and induce different biological functions upon CD154 binding. APC: antigen presenting cells, DC: dendritic cells, MC: monocyte, EC: endothelial cell, FB: fibroblast, SMC: smooth muscle cell, Mac: macrophage, ICAM: intercellular adhesion molecule, GPIb*α*: glycoprotein Ib*α*, JAM-3: junctional adhesion molecule-3, MPO: myeloperoxidase.

The *α*IIb*β*3 integrin, also known as GPIIb/IIIa, is a major platelet integrin of critical importance for platelet adhesion, aggregation, and thrombus formation. When activated by “inside out” signaling, in response to various platelet agonists (thrombin, collagen, ADP, or epinephrine), *α*IIb*β*3 will change its conformation allowing binding of its major ligands, including fibrinogen, fibronectin, and von Willebrand factor [30]. Binding is mediated through the RGD sequence found on many *α*IIb*β*3 ligands. Interestingly, CD154 was also found to interact with *α*IIb*β*3, primarily through the RGD sequence contained within the extracellular portion of the CD154 molecule. Binding of CD154 to *α*IIb*β*3 induces phosphorylation of tyrosine residues within the cytoplasmic domain of the *β*3 chain and appears necessary for stability of arterial thrombi [31]. These findings demonstrate that CD154, through its interaction with *α*IIb*β*3, is a platelet agonist that functions in an autocrine manner to regulate platelet biology (Figure 2).

### 3.3. *α*5*β*1

The *α*5*β*1 integrin has recently been identified as a novel functional receptor for CD154 [32]. Like *α*IIb*β*3, active *α*5*β*1 binds to its classical ligands fibrinogen and fibronectin through their RGD sequence [30]. Interestingly, we have previously shown that CD154 binds to inactive *α*5*β*1. In addition, we demonstrated that simultaneous binding of CD154 to CD40 and *α*5*β*1 is possible, indicating that CD154 interacts with *α*5*β*1 outside the CD40-binding site. The binding of CD154 to a monocytic cell line expressing *α*5*β*1 leads to the phosphorylation of the extracellular signal regulated kinase 1/2 (ERK1/2) and the expression of IL-8 mRNA in these cells, which is indicative of a functional consequence of the CD154/*α*5*β*1 interaction [32] (Figure 2). Even though *α*5*β*1 is expressed on endothelial cells, smooth muscle cells, and platelets, its role upon interacting with CD154 in these cells has not been elucidated yet. 

### 3.4. *α*M*β*2 (Mac-1)

AlphaM*β*2 or Mac-1 (CD11b/CD18), a member of the integrin family mainly expressed on monocytes/macrophages and neutrophils, has also recently been identified as a CD154 receptor [19]. As with its classical ligands, such as C3bi [33], intracellular adhesion molecule-1 [34], fibrinogen [35], vitronectin [36], factor Xa [37, 38], heparin [39, 40], glycoprotein Ib*α* [41, 42], junctional adhesion molecule-3 [43], and lipoproteins [44], only the active conformation of Mac-1 can interact with CD154. This pleiotropic binding ensues Mac-1 a role in immune responses, coagulation, and inflammation [45]. Indeed, Zirlik et al. demonstrated that CD154 functionally enhances monocyte adhesion and migration via its binding with Mac-1 on monocytes. Interestingly, CD154 stimulation was shown to trigger Mac-1-dependent nuclear factor-kB (NFkB) activation and enhance myeloperoxidase release from monocytes [19] (Figure 2). In addition, the CD154/Mac-1 interaction may play a significant role in atherogenesis, since Mac-1 inhibition in the LDLR^*‒*/*‒*^ atherosclerosis mouse model attenuates arterial plaque development and lesional macrophage accumulation [19]. Furthermore, Li et al. showed that CD154 upregulates Mac-1 expression on neutrophils and enhances leukocyte recruitment and neointima formation after arterial injury in ApoE^*‒*/*‒*^ mice, another atherosclerosis-prone mouse model [46]. These findings unravel a novel mechanism by which CD154 contributes to inflammatory events at the level of vascular cells.

## 4. CD154 in Autoimmune Diseases

CD154 contributes to the potentiation of autoimmune diseases in which B and T cell activation plays a major role, such as SLE, RA, lupus nephritis, multiple sclerosis, and autoimmune diabetes [47]. Indeed, a role for CD154/CD40 interactions have been identified in the development of Type I diabetes [48]. Moreover, signaling through CD40 was shown to induce the production of inflammatory cytokines in human and nonhuman primate islet cells [49]. High expression of CD154^+^ cells was also detected in the brains of patients with multiple sclerosis [47]. In addition, patients with psoriatic arthritis were found to exhibit CD40 expression on keratinocytes and endothelial cells within psoriatic plaques and increased expression of CD154 on peripheral blood T cells [50–52]. Increased levels of soluble CD154 were also reported in patients with SLE, RA, and Sjorgren's disease, in association with disease activity [53]. In fact, the CD154/CD40 interaction triggers a series of immune responses contributing to T-cell-dependent immunity at different levels in these diseases [54]. First, CD154 signaling could disrupt negative selection in the thymus allowing escape of self-reactive T cells and thus failure of central tolerance. Second, aberrant CD154-dependent production of proinflammatory cytokines could direct the differentiation of T cells to Th17 cells, a process that is augmented by activation of antigen-presenting cell. Third, CD154 interactions could stimulate inflammatory chemokines and cytokines within the target tissue, which contribute to tissue damage and propagation of the inflammatory assault [49, 55–58]. 

This review will focus primarily on recent findings concerning the role of CD154 in SLE and RA. 

### 4.1. Systemic Lupus Erythematosus (SLE)

SLE is a multiorgan target autoimmune disease characterized by a defect in the innate and adaptive immune systems, in which B cells are at the center of the pathogenesis. Indeed, B cells, with the aid of CD4^+^ T cells, secrete autoantibodies, activate the complement system, and favor the production of cytokines and other mediators potentially involved in inflammation, tissue damage, and progression of the disease [59]. Central to all these processes is signaling events mediated by CD154 [60, 61]. 

#### 4.1.1. Role of CD154 in Pathogenesis of SLE

**Figure 3:**
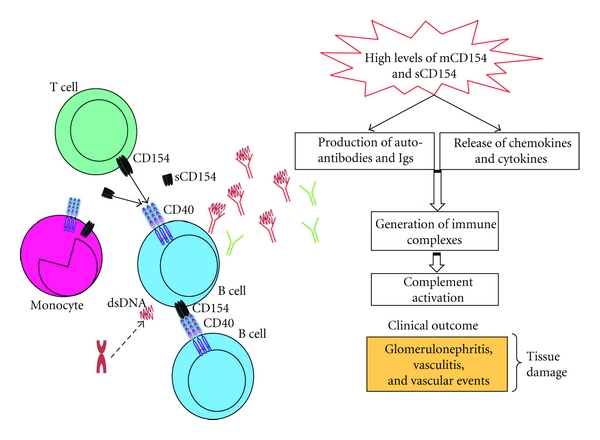
CD154-dependent mechanistic events in SLE patients. High levels of membrane bound and sCD154 activate various immune cells in SLE, including T cells, B cells, and monocytes. These CD154 interactions induce the release of inflammatory mediators and the production of autoantibody and Igs, leading to the generation of immune complexes and the activation of the complement system at the forefront of many clinical manifestations in SLE patients.

CD154 is overexpressed on T cells and atypically expressed on B cells and monocytes in patients with active SLE [62–64]. Ectopic expression of CD154 on B cells is also observed in lupus-prone BXSB mice [65]. Higushi et al. demonstrated that CD154-transgenic mice spontaneously produce autoantibodies such as anti-DNA Abs and develop lupus like glomerulonephritis with age [66]. Immunohistochemical analysis of CD154 expression in the biopsies of lupus kidney specimens showed an upregulation of CD154 expression on renal endothelial and tubular cells, and on interstitial infiltrating T cells [60]. Moreover, CD154 was shown to contribute to SLE pathogenesis by inducing the production of various chemokines in renal endothelial and tubular cells, thereby, increasing local inflammatory responses [67, 68]. The abnormally prolonged expression of CD154 on T cells and high levels of circulating sCD154 can activate bystander autoimmune B cells and initiate autoantibody secretion in SLE (Figure 3). Moreover, the enhanced CD154 expression on activated T cells is implicated in the overexpression of costimulatory molecules such as CD86 on B cells isolated from SLE patients. CD154-induced overexpression of CD86 is essential for anti-DNA antibody production in these patients [69]. In addition, it has been suggested that the atherosclerotic complications seen in patients with SLE are mediated by CD154 and its receptors [70, 71]. In fact, CD154 on activated platelets derived from patients with SLE can upregulate the expression of CD40 on mesangial cells and induce the release of soluble CD40. Such CD154-mediated responses activate mesangial cells, thus, stimulating their proliferation and production of TGF-*β*1 [72]. Interestingly, these responses are associated with glomerular injury and glomerulosclerosis in SLE, respectively. 

#### 4.1.2. Serum Levels of CD154 in SLE Patients

Circulating levels of sCD154 in patients with SLE correlate with the titers of anti- double-stranded DNA (dsDNA) autoantibodies and with disease activity [73, 74]. Soluble CD154, at concentrations reported in some SLE sera, is capable of inducing the upregulation of several accessory molecules on B cells, which favors B cell survival and differentiation, thereby, exacerbating the immune response [74]. Moreover, SLE patients exhibiting antiphospholipid antibodies in their sera and presenting with a history of thrombosis have higher circulating sCD154 levels than SLE-antiphospholipid antibody-positive patients with no history of thrombosis [75]. These data further support a possible role for the CD154 axis in the increased vascular events seen in SLE. Moreover, Aleksandrova et al. showed that enhanced levels of sCD154 in patients with SLE and secondary antiphospholipid syndrome are associated with pronounced intima-media thickness of the carotid arteries, hypercholesterinemia, and diastolic dysfunction [76]. These findings also support the association of CD154 with cardiovascular abnormalities in SLE.

#### 4.1.3. Anti-CD154 Treatment in SLE

In view of all the suggested roles of CD154 in SLE, many approaches using CD154 blocking monoclonal antibodies (mAbs) have been tested in murine models of the disease and resulted in positive outcomes. Treatment with anti-CD154 mAbs prior to disease onset prevents proteinuria, prolongs survival, ameliorates or even prevents kidney disease, and decreases anti-DNA autoantibody titers in the New Zealand Black/New Zealand White F_1_ systemic lupus erythematosus (NZBxNZW) F1 and (SWRxNZB) F1 mice models. Moreover, anti-CD154 treatment when nephritis has already developed still slows disease progression, reverses proteinuria, and induces remissions in mice, despite ongoing renal immune complex deposition. Responding mice show rapid downregulation of TNF-*α*, IL-10, and TGF-*β* mRNA levels [77, 78]. In addition, a short-term combination therapy with the costimulatory antagonists CTLA4-Ig and anti-CD154 in NZB/NZW F_1 _mice delays the onset of renal dysfunction, significantly decreases the frequency of B cells producing anti-DNA IgGs, partially suppresses class switching, and inhibits T cell activation and switching to memory phenotypes [79]. 

To date, two clinical studies investigating the use of anti-CD154 mAbs for the treatment of SLE have been conducted. In clinical trials, ruplizumab (BG9588) showed good clinical and laboratory responses in some SLE patients. However, the study was stopped earlier than expected because of thromboembolic events [80]. Toralizumab (IDEC-131) was tested in a double-blind, placebo-controlled study in patients with mild-to-moderately active SLE over 16 weeks. although the systemic lupus erythematosus disease activity index (SLEDAI) scores improved from the baseline levels of disease activity in all groups, these scores were not statistically significant among the IDEC-131 treated and placebo groups [61]. New reagents inhibiting CD154-mediated events without increasing the risk of thromboembolic complications are in development [81]. It is important to note at this level that developing new CD154 interfering agents should take into account the new receptors identified for CD154 [1]. 

### 4.2. Rheumatoid Arthritis (RA)

RA is a chronic, progressive, and debilitating autoimmune disease that occurs in approximately 1% of adults. A vicious cycle of inflammation and cartilage destruction is at the hallmark of the ongoing autoimmune reactions in the synovial tissue. Synovial inflammation, pannus formation, neoangiogenesis, and destruction of joint cartilage are mediated by the constant synthesis of matrix metalloproteinases (MMPs) and proinflammatory cytokines, such as TNF-*α* and IL-1. The infiltrating leukocytes and synovial cells destroy the cartilage tissue and erode bones, thereby, resulting in the loss of articular surfaces and joint motion [82]. 

#### 4.2.1. Role of CD154 in the Pathogenesis of RA

**Figure 4:**
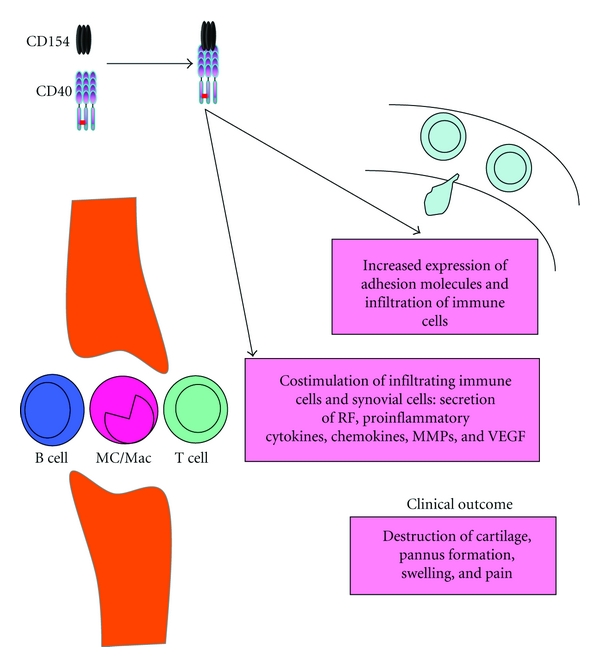
Biological role of CD154 in RA. CD154 contributes to the pathogenesis of RA by stimulating expression of adhesion molecules, production of RF, release of inflammatory mediators including cytokines, chemokines, MMPs, and others.

Many reports have demonstrated the implication of CD154 and CD40 in the pathogenesis of RA. In fact, CD154 is implicated in all pathogenic events of RA that ultimately lead to cartilage destruction and bone erosion. These include the T cell-mediated response, the presence of rheumatoid factors (RFs), the expression of adhesion molecules, synovial hyperplasia, and pannus formation, as well as the secretion of proinflammatory cytokines and MMPs (Figure 4). The enhanced expression of CD154 on T cells supports the theory of a T-cell-driven disease. CD154 mRNA and protein were shown to be upregulated on peripheral blood and synovial fluid T cells from RA patients, in comparison to control patients [83]. In addition, CD154 as well as CD40 are overexpressed by CD4^+^ and CD8^+^ T lymphocytes and macrophages of the synovial fluid of rheumatoid patients. This aberrant expression was postulated to contribute to the development of synovial hyperplasia [84, 85]. It was also suggested that the increased and prolonged expression of CD154 on T cells from RA patients might be contributing to enhanced cell function and articular inflammation [85, 86]. 

The inflammatory process in RA is dependent on both humoral- and cell-mediated immunity. CD154 on activated T cells is linked to RF synthesis [87], B cell-dependent IgG overproduction, and secretion of IL-12 by synovial dentritic cells and macrophages [85, 88, 89]. On the other hand, CD154 expressed on T lymphocytes induces other critical costimulatory molecules, namely, CD80 and CD86 on B cells, macrophages, and dendritic cells, which in turn can favor T cell activation. Activated T cells can then initiate specific cellular immune responses, through the secretion of proinflammatory cytokines. For instance, the CD154/CD40 interaction was shown to increase the production of TNF-*α*, which plays a major role in the pathogenesis of RA. Indeed, Harigai et al. demonstrated that ligation of CD40 on freshly isolated synovial cells, using a recombinant sCD154 protein, induced TNF-*α* and IL-1*β* production, a response further amplified by IFN*γ* [88]. 

Other investigators have also demonstrated that ligation of CD40 on CD68^+^ synovial macrophage cells as well as fibroblast-like synoviocytes enhances the expression of CD154, CD106, IL-6, stromal cell-derived factor 1, vascular endothelial cell growth factor (VEGF), IL-8, and regulated upon activation normal T-cell expressed and secreted (RANTES) [84, 90–95]. In addition, ligation of CD40 on synovial fibroblasts from RA patients by T cell CD154 was shown to stimulate neovascularization at the site of synovitis by enhancing VEGF protein and mRNA levels [96]. Moreover, the CD154/CD40 axis is directly linked to the inflammatory process by increasing the expression of important adhesion molecules on fibroblastic cells, such as E selectin, vascular cell adhesion molecule-1 (VCAM-1), and intercellular adhesion molecule-1 (ICAM-1). These responses thereby exacerbate the recruitment and infiltration of immune cells at the sites of inflammation [91]. CD154 further contributes to a deleterious degradative cycle in RA by inducing the expression and activation of MMPs (MMP-1, MMP-9, and MMP-3) [97, 98], which are well known to drive the degradation of extracellular matrix proteins in RA. 

Hence, it is now believed that CD154, through its involvement in the production of chemokines, cytokines, MMPs, adhesion molecules, and growth factors, contributes to pannus formation and perpetuation of inflammation in RA (Figure 4). It is also worth noting, that the CD154/CD40-dependent induction of inflammatory mediators by RA synovial cells is mediated via the activation of MAPKs, especially ERK-1/2, p38 and NF*κ*B [99].

#### 4.2.2. Serum Levels of CD154 in RA Patients

The importance of the CD154/CD40 axis in the pathophysiology of RA is further consolidated by the levels of circulating sCD154 found in patients. Indeed, high levels of sCD154 have been reported in most patients with juvenile idiopathic arthritis [100]. Moreover, serum levels of sCD154 are higher in patients with RA than in healthy subjects and significantly correlate with both IgM-RF and IgG-RF titers [101]. 

#### 4.2.3. Anti-CD154 Treatment in RA

CD154/CD40 signaling was demonstrated to be critical in the initiation and progression of the mouse collagen-induced arthritis (CIA) model. Treatment of mice with agonistic anti-CD40 Abs at the time of CIA induction exacerbates the disease [102]. Conversely, the administration of antagonistic anti-CD154 mAbs prior to induction of CIA significantly ameliorates the disease [103], as manifested by the inhibition of symptoms such as development of joint inflammation, serum antibody titers to collagen, infiltration of inflammatory cells into the subsynovial tissue, and erosion of cartilage and bone in treated mice [104]. Anti-CD154 treatment in the K/BxN arthritis mouse model was shown to induce prophylactic effects, as antibody administration inhibits development in mice when given before the onset of the clinically apparent disease [105]. However, anti-CD154 therapy has no effect when administered after clinical onset. These results support the CD154 axis as a therapeutic target in the treatment of RA.

## 5. Conclusions and Future Studies

The current review supports the role of CD154 as a major participant in the pathogenesis of autoimmune diseases, particularly RA and SLE. As previously detailed, the CD154 axis, through its diverse distribution on many cell types, has acquired pleiotropic functions and contributes to inflammatory diseases by triggering the secretion of critical inflammatory mediators potentially involved in tissue degradation and damage. It is now well established that CD154 interacts with many receptors, namely, CD40, *α*IIb*β*3, *α*5*β*1, and *α*M*β*2. For instance, it can constitutively bind to CD40 and *α*5*β*1, as well as the active forms of *α*IIb*β*3 and *α*M*β*2. Interestingly, CD154 can simultaneously bind to two receptors expressed on the same cell surface. This phenomenon may grant CD154 distinct biological activities, depending on the receptor in question and the signaling pathway it might trigger. Even though, most of the biological functions of CD154 are believed to involve its interaction with CD40, the discovery of other receptors for CD154 unravels new roles for this molecule in mediating immune and inflammatory events at the forefront of the pathogenesis of many diseases including autoimmune disorders. However, little is known about the signaling pathways and cellular responses triggered by the interaction of CD154 with its other receptors. Indeed, the elucidation of the precise molecular pathways induced by the CD154/*α*IIb*β*3, CD154/*α*M*β*2, and CD154/*α*5*β*1 interactions will allow a better understanding of the biological roles of CD154 in associated diseases and allow the design of better therapeutic strategies for the management of these disease conditions. 

## References

[1] Hassan GS, Merhi Y, Mourad WM (2009). CD154 and its receptors in inflammatory vascular pathologies. *Trends in Immunology*.

[2] Clark LB, Foy TM, Noelle RJ (1996). CD40 and its ligand. *Advances in Immunology*.

[3] Van Kooten C, Banchereau J (1996). CD40-CD40 ligand: a multifunctional receptor-ligand pair. *Advances in Immunology*.

[4] Grewal IS, Flavell RA (1998). CD40 and CD154 in cell-mediated immunity. *Annual Review of Immunology*.

[5] Locksley RM, Killeen N, Lenardo MJ (2001). The TNF and TNF receptor superfamilies: integrating mammalian biology. *Cell*.

[6] Schönbeck U, MacH F, Libby P (2000). CD154 (CD40 ligand). *International Journal of Biochemistry and Cell Biology*.

[7] Graf D, Korthauer U, Mages HW, Senger G, Kroczek RA (1992). Cloning of TRAP, a ligand for CD40 on human T cells. *European Journal of Immunology*.

[8] Villa A, Notarangelo LD, Di Santo JP (1994). Organization of the human CD40L gene: implications for molecular defects in X chromosome-linked hyper-IgM syndrome and prenatal diagnosis. *Proceedings of the National Academy of Sciences of the United States of America*.

[9] Van Kooten G, Banchereau J (2000). CD40-CD40 ligand. *Journal of Leukocyte Biology*.

[10] Fanslow WC, Srinivasan S, Paxton R, Gibson MG, Spriggs MK, Armitage RJ (1994). Structural characteristics of CD40 ligand that determine biological function. *Seminars in Immunology*.

[11] Singh J, Garber E, Van Vlijmen H (1998). The role of polar interactions in the molecular recognition of CD40L with its receptor CD40. *Protein Science*.

[12] An H-J, Kim YJ, Song DH (2011). Crystallographic and mutational analysis of the CD40-CD154 complex and its implications for receptor activation. *Journal of Biological Chemistry*.

[13] Henn V, Steinbach S, Büchner K, Presek P, Kroczek RA (2001). The inflammatory action of CD40 ligand (CD154) expressed on activated human platelets is temporally limited by coexpressed CD40. *Blood*.

[14] Schönbeck U, Libby P (2001). The CD40/CD154 receptor/ligand dyad. *Cellular and Molecular Life Sciences*.

[15] André P, Srinivasa Prasad KS, Denis CV (2002). CD40L stabilizes arterial thrombi by a *β*3 integrin-dependent mechanism. *Nature Medicine*.

[16] André P, Nannizzi-Alaimo L, Prasad SK, Phillips DR (2002). Platelet-derived CD40L: the switch-hitting player of cardiovascular disease. *Circulation*.

[17] Noelle RJ, Roy M, Shepherd DM, Stamenkovic I, Ledbetter JA, Aruffo A (1992). A 39-kDa protein on activated helper T cells binds CD40 and transduces the signal for cognate activation of B cells. *Proceedings of the National Academy of Sciences of the United States of America*.

[18] Léveillé C, Bouillon M, Guo W (2007). CD40 ligand binds to *α*5*β*1 integrin and triggers cell signaling. *Journal of Biological Chemistry*.

[19] Zirlik A, Maier C, Gerdes N (2007). CD40 ligand mediates inflammation independently of CD40 by interaction with Mac-1. *Circulation*.

[20] Lafage-Pochitaloff M, Herman P, Birg F (1994). Localization of the human CD40 gene to chromosome 20, bands q12-q13.2. *Leukemia*.

[21] Tone M, Tone Y, Fairchild PJ, Wykes M, Waldmann H (2001). Regulation of CD40 function by its isoforms generated through alternative splicing. *Proceedings of the National Academy of Sciences of the United States of America*.

[22] Naismith JH, Sprang SR (1998). Modularity in the TNF-receptor family. *Trends in Biochemical Sciences*.

[23] Reyes-Moreno C, Sharif-Askari E, Girouard J (2007). Requirement of oxidation-dependent CD40 homodimers for CD154/CD40 bidirectional signaling. *Journal of Biological Chemistry*.

[24] Bock J, Gulbins E (2003). The transmembranous domain of CD40 determines CD40 partitioning into lipid rafts. *FEBS Letters*.

[25] Braesch-Andersen S, Paulie S, Koho H, Nika H, Aspenstrom P, Perlmann P (1989). Biochemical characteristics and partial amino acid sequence of the receptor-like human B cell and carcinoma antigen CDw40. *Journal of Immunology*.

[26] Chan FKM, Chun HJ, Zheng L, Siegel RM, Bui KL, Lenardo MJ (2000). A domain in TNF receptors that mediates ligand-independent receptor assembty and signaling. *Science*.

[27] Mukundan L, Milhorn DM, Matta B, Suttles J (2004). CD40-mediated activation of vascular smooth muscle cell chemokine production through a Src-initiated, MAPK-dependent pathway. *Cellular Signalling*.

[28] Chen C, Chai H, Wang X (2008). Soluble CD40 ligand induces endothelial dysfunction in human and porcine coronary artery endothelial cells. *Blood*.

[29] Dongari-Bagtzoglou AI, Thienel U, Yellin MJ (2003). CD40 ligation triggers COX-2 expression in endothelial cells: evidence that CD40-mediated IL-6 synthesis is COX-2-dependent. *Inflammation Research*.

[30] Hynes RO (2002). Integrins: bidirectional, allosteric signaling machines. *Cell*.

[31] Prasad KSS, Andre P, He M, Bao M, Manganello J, Phillips DR (2003). Soluble CD40 ligand induces *β*3 integrin tyrosine phosphorylation and triggers platelet activation by outside-in signaling. *Proceedings of the National Academy of Sciences of the United States of America*.

[32] Léveillé C, Bouillon M, Guo W (2007). CD40 ligand binds to *α*5*β*1 integrin and triggers cell signaling. *Journal of Biological Chemistry*.

[33] Ross GD, Lambris JD (1982). Identification of a C3bi-specific membrane complement receptor that is expressed on lymphocytes, monocytes, neutrophils, and erythrocytes. *Journal of Experimental Medicine*.

[34] Diamond MS, Staunton DE, Marlin SD, Springer TA (1991). Binding of the integrin Mac-1 (CD11b/CD18) to the third immunoglobulin-like domain of ICAM-1 (CD54) and its regulation by glycosylation. *Cell*.

[35] Altieri DC, Agbanyo FR, Plescia J, Ginsberg MH, Edgington TS, Plow EF (1990). A unique recognition site mediates the interaction of fibrinogen with the leukocyte integrin Mac-1 (CD11b/CD18). *Journal of Biological Chemistry*.

[36] Kanse SM, Matz RL, Preissner KT, Peter K (2004). Promotion of leukocyte adhesion by a novel interaction between vitronectin and the *β*2 integrin Mac-1 (*α*M*β* 2, CD11b/CD18). *Arteriosclerosis, Thrombosis, and Vascular Biology*.

[37] Altieri DC, Edgington TS (1988). The saturable high affinity association of factor X to ADP-stimulated monocytes defines a novel function of the Mac-1 receptor. *Journal of Biological Chemistry*.

[38] Schwarz M, Nordt T, Bode C, Peter K (2002). The GP IIb/IIIa inhibitor abciximab (c7E3) inhibits the binding of various ligands to the leukocyte integrin Mac-1 (CD11b/CD18, *α*M*β*2). *Thrombosis Research*.

[39] Diamond MS, Alon R, Parkos CA, Quinn MT, Springer TA (1995). Heparin is an adhesive ligand for the leukocyte integrin Mac-1 (CD11b/CD18). *Journal of Cell Biology*.

[40] Peter K, Schwarz M, Conradt C (1999). Heparin inhibits ligand binding to the leukocyte integrin Mac-1 (CD11b/CD18). *Circulation*.

[41] Simon DI, Chen Z, Xu H (2000). Platelet glycoprotein Ib*α* is a counterreceptor for the leukocyte integrin Mac-1 (CD11b/CD18). *Journal of Experimental Medicine*.

[42] Ehlers R, Ustinov V, Chen Z (2003). Targeting platelet-leukocyte interactions: identification of the integrin Mac-1 binding site for the platelet counter receptor glycoprotein Ib*α*. *Journal of Experimental Medicine*.

[43] Santoso S, Sachs UJH, Kroll H (2002). The junctional adhesion molecule 3 (JAM-3) on human platelets is a counterreceptor for the leukocyte integrin Mac-1. *Journal of Experimental Medicine*.

[44] Sotiriou SN, Orlova VV, Al-Fakhri N (2006). Lipoprotein(a) in atherosclerotic plaques recruits inflammatory cells through interaction with Mac-1 integrin. *FASEB Journal*.

[45] Anderson DC, Rothlein R, Marlin SD, Krater SS, Smith CW (1990). Impaired transendothelial migration by neonatal neutrophils: abnormalities of Mac-1 (CD11b/CD18)-dependent adherence reactions. *Blood*.

[46] Li G, Sanders JM, Bevard MH (2008). CD40 ligand promotes Mac-1 expression, leukocyte recruitment, and neointima formation after vascular injury. *American Journal of Pathology*.

[47] Gerritse K, Laman JD, Noelle RJ (1996). CD40-CD40 ligand interactions in experimental allergic encephalomyelitis and multiple sclerosis. *Proceedings of the National Academy of Sciences of the United States of America*.

[48] Balasa B, Krahl T, Patstone G (1997). CD40 ligand-CD40 interactions are necessary for the initiation of insulitis and diabetes in nonobese diabetic mice. *Journal of Immunology*.

[49] Barbé-Tuana FM, Klein D, Ichii H (2006). CD40-CD40 ligand interaction activates proinflammatory pathways in pancreatic islets. *Diabetes*.

[50] Denfeld RW, Hollenbaugh D, Fehrenbach A (1996). CD40 is functionally expressed on human keratinocytes. *European Journal of Immunology*.

[51] Ohta Y, Hamada Y (2004). In situ expression of CD40 and CD40 ligand in psoriasis. *Dermatology*.

[52] Daoussis D, Antonopoulos I, Andonopoulos AP, Liossis SNC (2007). Increased expression of CD154 (CD40L) on stimulated T-cells from patients with psoriatic arthritis. *Rheumatology*.

[53] Toubi E, Shoenfeld Y (2004). The role of CD40-CD154 interactions in autoimmunity and the benefit of disrupting this pathway. *Autoimmunity*.

[54] Peters AL, Stunz LL, Bishop GA (2009). CD40 and autoimmunity: the dark side of a great activator. *Seminars in Immunology*.

[55] Akiyama T, Shimo Y, Yanai H (2008). The tumor necrosis factor family receptors RANK and CD40 cooperatively establish the thymic medullary microenvironment and self-tolerance. *Immunity*.

[56] Iezzi G, Sonderegger I, Ampenberger F, Schmitz N, Marsland BJ, Kopf M (2009). CD40-CD40L cross-talk integrates strong antigenic signals and microbial stimuli to induce development of IL-17-producing CD4+ T cells. *Proceedings of the National Academy of Sciences of the United States of America*.

[57] Bottazzo GF, Pujol Borrell R, Hanafusa T, Feldmann M (1983). Role of aberrant HLA-DR expression and antigen presentation in induction of endocrine autoimmunity. *Lancet*.

[58] Jacobson EM, Huber AK, Akeno N (2007). A CD40 Kozak sequence polymorphism and susceptibility to antibody-mediated autoimmune conditions: the role of CD40 tissue-specific expression. *Genes and Immunity*.

[59] Gualtierotti R, Biggioggero M, Penatti AE, Meroni PL (2010). Updating on the pathogenesis of systemic lupus erythematosus. *Autoimmunity Reviews*.

[60] Yellin MJ, Thienel U (2000). T cells in the pathogenesis of systemic lupus erythematosus: potential roles of CD154-CD40 interactions and costimulatory molecules. *Current Rheumatology Reports*.

[61] Hassan GS (2009). Implication of CD154/CD40 interaction in healthy and autoimmune responses. *Current Immunology Reviews*.

[62] Desai-Mehta A, Lu L, Ramsey-Goldman R, Datta SK (1996). Hyperexpression of CD40 ligand by B and T cells in human lupus and its role in pathogenic autoantibody production. *Journal of Clinical Investigation*.

[63] Koshy M, Berger D, Crow MK (1996). Increased expression of CD40 ligand on systemic lupus erythematosus lymphocytes. *Journal of Clinical Investigation*.

[64] Katsiari CG, Liossis SNC, Souliotis VL, Dimopoulos AM, Manoussakis MN, Sfikakis PP (2002). Aberrant expression of the costimulatory molecule CD40 ligand on monocytes from patients with systemic lupus erythematosus. *Clinical Immunology*.

[65] Henn V, Slupsky JR, Gräfe M (1998). CD40 ligand on activated platelets triggers an inflammatory reaction of endothelial cells. *Nature*.

[66] Higuchi T, Aiba Y, Nomura T (2002). Cutting edge: ectopic expression of CD40 ligand on B cells induces lupus-like autoimmune disease. *Journal of Immunology*.

[67] Yellin MJ, D’Agati V, Parkinson G (1997). Immunohistologic analysis of renal CD40 and CD40L expression in lupus nephritis and other glomerulonephritides. *Arthritis and Rheumatism*.

[68] Van Kooten C, Gerritsma JSJ, Paape ME, Van Es LA, Banchereau J, Daha MR (1997). Possible role for CD40-CD40L in the regulation of interstitial infiltration in the kidney. *Kidney International*.

[69] Nagafuchi H, Shimoyama Y, Kashiwakura J, Takeno M, Sakane T, Suzuki N (2003). Preferential expression of B7.2 (CD86), but not B7.1 (CD80), on B cells induced by CD40/CD40L interaction is essential for anti-DNA autoantibody production in patients with systemic lupus erythematosus. *Clinical and Experimental Rheumatology*.

[70] Abou-Raya A, Abou-Raya S (2006). Inflammation: a pivotal link between autoimmune diseases and atherosclerosis. *Autoimmunity Reviews*.

[71] Rhew EY, Ramsey-Goldman R (2006). Premature atherosclerotic disease in systemic lupus erythematosus—Role of inflammatory mechanisms. *Autoimmunity Reviews*.

[72] Delmas Y, Viallard JF, Solanilla A (2005). Activation of mesangial cells by platelets in systemic lupus erythematosus via a CD154-dependent induction of CD40. *Kidney International*.

[73] Kato K, Santana-Sahagún E, Rassenti LZ (1999). The soluble CD40 ligand sCD154 in systemic lupus erythematosus. *Journal of Clinical Investigation*.

[74] Vakkalanka RK, Woo C, Kirou KA, Koshy M, Berger D, Crow MK (1999). Elevated levels and functional capacity of soluble CD40 ligand in systemic lupus erythematosus sera. *Arthritis and Rheumatism*.

[75] Ferro D, Pignatelli P, Loffredo L (2004). Soluble CD154 plasma levels in patients with systemic lupus erythematosus: modulation by antiphospholipid antibodies. *Arthritis and Rheumatism*.

[76] Aleksandrova EN, Novikov AA, Popkova TV (2006). Soluble CD40 ligand in systemic lupus erythematosus and antiphospholipid syndrome. *Terapevticheskii Arkhiv*.

[77] Quezada SA, Eckert M, Adeyi OA, Schned AR, Noelle RJ, Burns CM (2003). Distinct mechanisms of action of anti-CD154 in early versus late treatment of murine lupus nephritis. *Arthritis and Rheumatism*.

[78] Wang X, Huang W, Schiffer LE (2003). Effects of anti-CD154 treatment on B cells in murine systemic lupus erythematosus. *Arthritis and Rheumatism*.

[79] Wang X, Huang W, Mihara M, Sinha J, Davidson A (2002). Mechanism of action of combined short-term CTLA4Ig and anti-CD40 ligand in murine systemic lupus erythematosus. *Journal of Immunology*.

[80] Boumpas DT, Furie R, Manzi S (2003). A short course of BG9588 (anti-CD40 ligand antibody) improves serologic activity and decreases hematuria in patients with proliferative lupus glomerulonephritis. *Arthritis and Rheumatism*.

[81] Yildirim-Toruner C, Diamond B (2011). Current and novel therapeutics in the treatment of systemic lupus erythematosus. *Journal of Allergy and Clinical Immunology*.

[82] McInnes IB, O’Dell JR (2010). State-of-the-art: rheumatoid arthritis. *Annals of the Rheumatic Diseases*.

[83] MacDonald KPA, Nishioka Y, Lipsky PE, Thomas R (1997). Functional CD40 ligand is expressed by T cells in rheumatoid arthritis. *Journal of Clinical Investigation*.

[84] Rissoan MC, Van Kooten C, Chomarat P (1996). The functional CD40 antigen of fibroblasts may contribute to the proliferation of rheumatoid synovium. *Clinical and Experimental Immunology*.

[85] Liu MF, Chao SC, Wang CR, Lei HY (2001). Expression of CD40 and CD40 ligand among cell populations within rheumatoid synovial compartment. *Autoimmunity*.

[86] Reparon-Schuijt CC, Van Esch WJE, Van Kooten C (2001). Secretion of anti-citrulline-containing peptide antibody by B lymphocytes in rheumatoid arthritis. *Arthritis and Rheumatism*.

[87] Kyburz D, Corr M, Brinson DC, Von Damm A, Tighe H, Carson DA (1999). Human rheumatoid factor production is dependent on CD40 signaling and autoantigen. *Journal of Immunology*.

[88] Harigai M, Hara M, Nakazawa S (1999). Ligation of CD40 induced tumor necrosis factor-*α* in rheumatoid arthritis: a novel mechanism of activation of synoviocytes. *Journal of Rheumatology*.

[89] Kitagawa M, Mitsui H, Nakamura H (1999). Differential regulation of rheumatoid synovial cell interleukin-12 production by tumor necrosis factor *α* and CD40 signals. *Arthritis and Rheumatism*.

[90] Kitagawa M, Suzuki H, Adachi Y, Nakamura H, Yoshino S, Sumida T (2001). Interferon-*γ* enhances interleukin 12 production in rheumatoid synovial cells via CD40-CD154 dependent and independent pathways. *Journal of Rheumatology*.

[91] Yellin MJ, Winikoff S, Fortune SM (1995). Ligation of CD40 on fibroblasts induces CD54 (ICAM-1) and CD106 (VCAM-1) up-regulation and IL-6 production and proliferation. *Journal of Leukocyte Biology*.

[92] Nanki T, Hayashida K, El-Gabalawy HS (2000). Stromal cell-derived factor-1-CXC chemokine receptor 4 interactions play a central role in CD4+ T cell accumulation in rheumatoid arthritis synovium. *Journal of Immunology*.

[93] Cho CS, Cho ML, Min SY (2000). CD40 engagement on synovial fibroblast up-regulates production of vascular endothelial growth factor. *Journal of Immunology*.

[94] Kornbluth RS, Kee K, Richman DD (1998). CD40 ligand (CD154) stimulation of macrophages to produce HIV-1-suppressive *β*-chemokines. *Proceedings of the National Academy of Sciences of the United States of America*.

[95] McDyer JF, Dybul M, Goletz TJ (1999). Differential effects of CD40 ligand/trimer stimulation on the ability of dendritic cells to replicate and transmit HIV infection: evidence for CC- chemokine-dependent and -independent mechanisms. *Journal of Immunology*.

[96] Cho CS, Cho ML, Min SY (2000). CD40 engagement on synovial fibroblast up-regulates production of vascular endothelial growth factor. *Journal of Immunology*.

[97] Malik N, Greenfield BW, Wahl AF, Kiener PA (1996). Activation of human monocytes through CD40 induces matrix metalloproteinases. *Journal of Immunology*.

[98] Mach F, Schönbeck U, Bonnefoy JY, Pober JS, Libby P (1997). Activation of monocyte/macrophage functions related to acute atheroma complication by ligation of CD40: induction of collagenase, stromelysin, and tissue factor. *Circulation*.

[99] Harigai M, Hara M, Kawamoto M (2004). Amplification of the synovial inflammatory response through activation of mitogen-activated protein kinases and nuclear factor *κ*B using ligation of CD40 on CD14+ synovial cells from patients with rheumatoid arthritis. *Arthritis and Rheumatism*.

[100] Prahalad S, Martins TB, Tebo AE (2008). Elevated serum levels of soluble CD154 in children with juvenile idiopathic arthritis. *Pediatric Rheumatology*.

[101] Tamura N, Kobayashi S, Kato K (2001). Soluble CD154 in rheumatoid arthritis: elevated plasma levels in cases with vasculitis. *Journal of Rheumatology*.

[102] Tellander AC, Michaëlsson E, Brunmark C, Andersson M (2000). Potent adjuvant effect by anti-CD40 in collagen-induced arthritis. Enhanced disease is accompanied by increased production of collagen type-II reactive IgG2a and IFN-*γ*. *Journal of Autoimmunity*.

[103] Li L, Wang H, Wang B (2008). Anergic cells generated by blocking CD28 and CD40 costimulatory pathways in vitro ameliorate collagen induced arthritis. *Cellular Immunology*.

[104] Durie FH, Fava RA, Foy TM, Aruffo A, Ledbetter JA, Noelle RJ (1993). Prevention of collagen-induced arthritis with an antibody to gp39, the ligand for CD40. *Science*.

[105] Kyburz D, Carson DA, Corr M (2000). The role of CD40 ligand and tumor necrosis factor *α* signaling in the transgenic K/BxN mouse model of rheumatoid arthritis. *Arthritis and Rheumatism*.

